# Air Curtains Equipped With Hydroalcoholic Aerosol Sprayers for Massive COVID-19 Disinfection

**DOI:** 10.3389/fpubh.2020.582782

**Published:** 2021-01-28

**Authors:** Judit Raventós, Raimon Sabate

**Affiliations:** ^1^Service of Psychiatry, Psychology and Psychosomatic Medicine, Dexeus University Hospital, Barcelona, Spain; ^2^Department of Pharmacy and Pharmaceutical Technology and Physical-Chemistry, School of Pharmacy and Food Sciences, University of Barcelona, Barcelona, Spain; ^3^Institute of Nanoscience and Nanotechnology (IN2UB), Barcelona, Spain

**Keywords:** COVID-19, SARS-CoV2, coronaviral infections, viral outbreak investigation, pandemic (COVID-19), SARS-CoV2 infection

Coronavirus disease 2019 (COVID-19) affects thousands of healthcare workers in Europe. Latest figures show that healthcare workers represent up to 9 and 14% of Italy's and Spain's COVID-19 cases, respectively. In China, more than 3,300 healthcare workers have been contaminated (1, 2). Better protection for healthcare workers is clearly required. High levels of COVID-19 contamination in specialist wards and intensive care units and of equipment (from keyboards to gel hand sanitizers) indicate that the measures currently taken to control COVID-19 are insufficient, especially in hospital centers where a large number of infections occur and where the most vulnerable population is found.

COVID-19 incubation after a person-to-person transmission is stated to be of 2–10 days (about 5 days in average). The virus can spread *via* droplets falling on surfaces, e.g., clothing, and subsequent transfer *via* people's hands. It has been shown that coronaviruses such as severe acute respiratory syndrome (SARS), Middle East respiratory syndrome (MERS), or endemic human coronavirus (HCoV) can persist on inanimate surfaces for up to 9 days (3). Remarkably, surface disinfection with 0.1% sodium hypochlorite or 62–71% ethanol significantly reduces coronaviruses' infectivity after only 1 min of exposure. Since the stability of COVID-19 is similar to that of SARS-CoV-1 under analogous experimental circumstances (4), a potentially similar effect of these disinfectants against COVID-19 can be expected (3, 5). Hence, the implementation of hydroalcoholic disinfection firewalls as complementary disinfection systems for areas prone to having high viral loads, such as waiting rooms, entrances of hospital centers, etc., would minimize potential transmissions.

Due to the current lack of an efficient therapy or vaccine against COVID-19, the early containment to prevent the spread and the use of palliative/symptomatic treatments represent the two key options to slow down both the transmission (total confirmed cases) and mortality (total deaths) (6, 7). Most affected countries have opted, to a greater or lesser extent, for a combined system of global population confinement and treatment of the most serious cases (6, 9). In contrast, the UK has chosen not to confine the entire population and protect populations at risk (8). South Korea preferred the massive use of tests to control the spread (9).

The containment measures recommended by the WHO are aimed at avoiding person-to-person transmission; protective measures such as wearing masks and hand washing are meant to reduce the spread of coronaviruses through droplets (7). It should be taken into account that not all people will clean their hands correctly or will simply not do it. Furthermore, COVID-19 is stable in aerosols for hours and for days on surfaces, persisting for up to 9 days in the air (4), and on inanimate surfaces like metal, wood, paper, glass, plastic, ceramic, Teflon, polyvinyl chloride (PVC), silicon rubber, surgical gloves (latex), clothing (3), as well as on skin (such as face or hands). Considering these facts, current disinfection protocols are most likely insufficient to effectively eliminate viral presence in the air and on clothing and surfaces. The need to settle an effective massive disinfection system is evident.

As evidenced in Figure 1, both the total confirmed cases and total deaths augment linearly, indicating that the control of the COVID-19 outbreak will need some time. The number of new deaths per day appears to be stabilized around WHO situation report 70; comparison of the data between March 30, 2020, and August 7, 2020 (Figures 1B,D), suggests that early containment/prevention of further spread and palliative/symptomatic treatments are effective measures. Importantly, new clinical treatments using potential drugs like dexamethasone have allowed stabilizing the number of new deaths per day. In contrast, the increase of new confirmed cases per day worldwide suggests that the current (local) containment/prevention practices to contain COVID-19 spreading are completely insufficient. New and complementary methods for the disinfection/decontamination of COVID-19-infested environments are definitively required. For instance, recent data have revealed an exponential increase of new cases in territories where exceptional confinement measures have been relaxed, such as Japan or Hong Kong (7).

**Figure 1:**
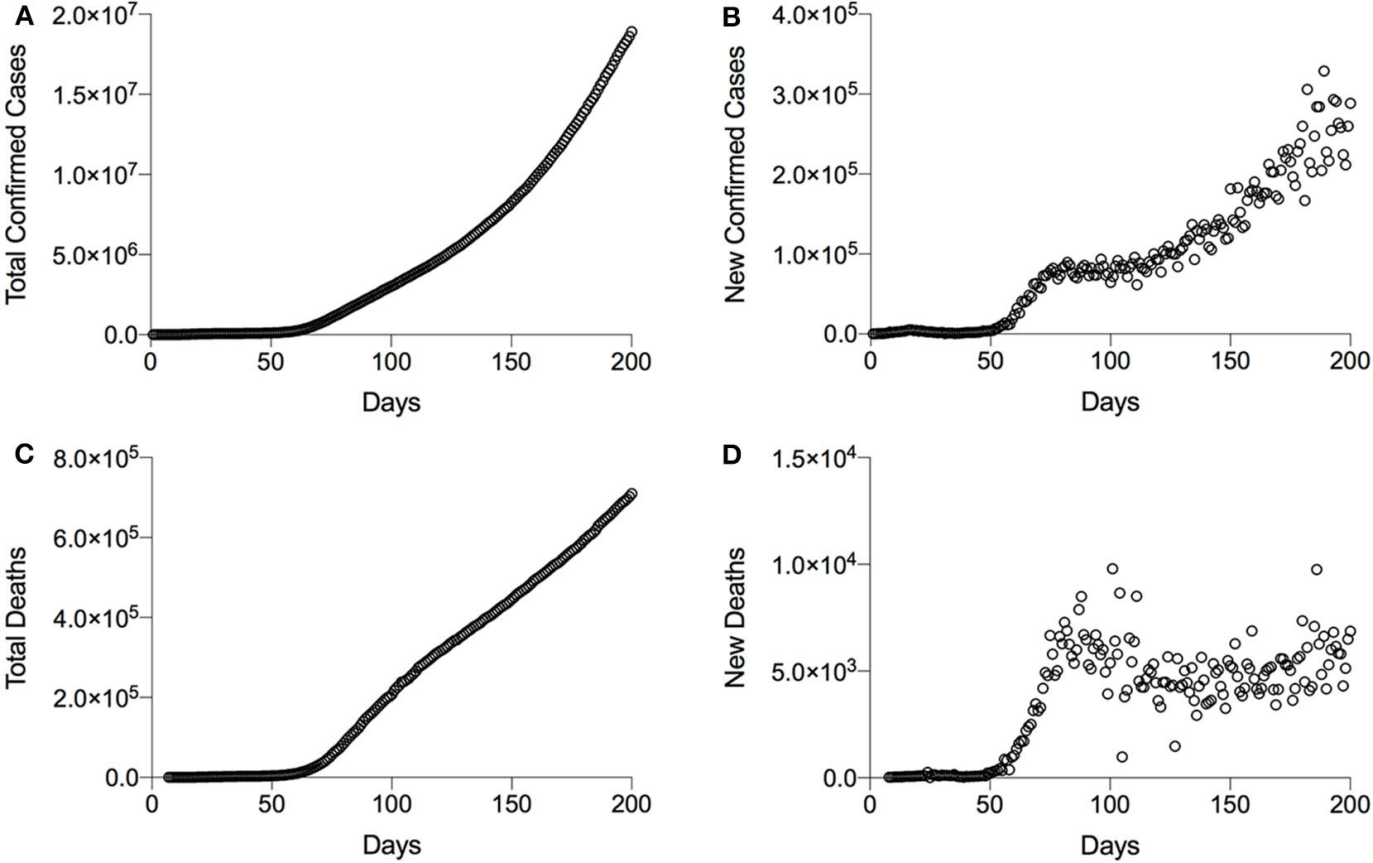
Graphical representation of the global evolution of coronavirus disease 2019 (COVID-19) transmission and mortality. **(A)** Representation of the transmission as a function of total confirmed cases per day. **(B)** New cases per day. **(C)** Representation of the mortality as a function of total deaths per day. **(D)** New deaths per day. ^*^Data are obtained from WHO situation reports; the first situation report was published on January 20, 2020. ^**^WHO situation report number 28 gives all confirmed cases including both laboratory-confirmed and clinically diagnosed cases. To normalize the data, the total number of confirmed cases obtained from WHO reports 1 and 27 has been mathematically predicted, taking into account the clinically diagnosed cases. This adjustment has only been necessary in the case of China.

In summary, there is an urgent need to find a highly efficient procedure to disinfect exposed body parts, clothing, and surfaces, which should be inexpensive and easy to implement. Since 62–71% hydroethanolic solutions significantly reduce COVID-19 infectivity within 1 min of exposure (3), we propose to equip air curtains with hydroalcoholic (70% ethanol) aerosol sprayers (or directly installing hydroalcoholic aerosol sprayers) in hospitals and healthcare centers. Nebulizers of hydroalcoholic aerosols may also be installed in supermarkets, malls, and restaurants and in any other overcrowded place such as stadiums, concert halls, etc. It has been shown that the contact of 70% hydroalcoholic mixtures with the skin is not detrimental to health, and that such solutions do not cause damages on inert surfaces or tissues. Importantly, it has recently been demonstrated that the lifetimes at 25°C of hydroalcoholic droplets containing 0:100, 50:50, and 100:0 ethanol:water mixtures are 1,488, 1,035, and 183 s, respectively; hence, a theoretical lifetime of ~641 s for 70% hydroalcoholic droplets can be calculated by lineal regression with *r*^2^ = 0.9698 (10). Thus, the lifetime of hydroalcoholic droplets, which is 10 times longer than the active infectious period of the coronavirus, should be enough to remove all traces of COVID-19. The evaporation of the droplets is crucial regarding the effectiveness of the treatment and could be a handicap for the implementation of this disinfection system. The humidity level and the ambient temperature are key factors for the maintenance of the hydroalcoholic drops over time. Therefore, this might be a limiting factor in warm climates and/or under low relative humidity. However, the high versatility and adaptability of the system would allow these handicaps to be successfully addressed; for example, a simple change of the type of spray nozzles can modify the size of the droplets and thus modify their lifetime.

Potential over-decontamination and low environmental impact or toxicity to people or animals would be minimal because the hydroalcoholic solutions will be applied in very specific and limited areas (*viz*. the passage areas of the air curtains) and for very short periods of time. Furthermore, it has been shown that the quantity of ethanol absorbed after intensive hand disinfection using commercially available hand rubs is minimal and below human toxic levels (11). Thus, 60–95% ethanol solutions are considered safe and effective for topical use on hands (11, 12). Whereas spraying individuals with disinfectants such as formaldehyde, chlorine-based agents, quaternary ammonium compounds, or other toxic chemicals is not recommended and may cause eye and skin irritation, bronchospasm, and gastrointestinal effects (7, 13–15), sprays containing hydroalcoholic solutions are widely used in cosmetics (e.g., deodorants) and are considered safe even at the respiratory level since it depends on the respiratory minute volume (16); in the case of hand washing, respiratory levels are very low.

Air curtains equipped with sprayers would effectively distribute hydroalcoholic droplets to surfaces, e.g., clothing, exposed body parts, equipment, etc., passing through, hence reducing the virus load. The systematic installation of hydroalcoholic nebulizers to the existing air curtains can help, in addition to the WHO recommendations, to prevent the spread of the virus (1, 7); this would represent an additional and nondisruptive measure to rapidly and economically reduce the propagation of the virus. Although the application of the proposed system will not avoid in any case the spread of the virus by an infected person through droplets or contact, and that it will be impossible to access all potentially contaminated regions, the possibility to substantially reduce viral loads on clothing and visible parts of the body can be considerate as a real and hopeful advance in COVID-19 disinfection.

## Author Contributions

JR and RS contributed to the conceptualization, writing the original draft, and reviewing and editing. RS contributed to the supervision, project administration, and funding acquisition. Both authors have read and agreed to the published version of the manuscript.

## Conflict of Interest

The authors declare that the research was conducted in the absence of any commercial or financial relationships that could be construed as a potential conflict of interest.
